# Adjuvant glycerol is not beneficial in experimental pneumococcal meningitis

**DOI:** 10.1186/1471-2334-10-84

**Published:** 2010-03-30

**Authors:** Cornelia Blaser, Matthias Klein, Denis Grandgirard, Matthias Wittwer, Heikki Peltola, Michael Weigand, Uwe Koedel, Stephen L Leib

**Affiliations:** 1 Institute for Infectious Diseases, University of Bern, 3010 Bern, Switzerland; 2 Department of Neurology, Klinikum Großhadern, Ludwig-Maximilians University, 81377 Munich, Germany; 3 Helsinki University Central Hospital, Hospital for Children and Adolescents, 00029 HUS Helsinki, Finland; 4 Institute of Clinical Chemistry, Klinikum Großhadern, Ludwig-Maximilians University, 81377 Munich, Germany; 5 The Meningitis Study Group of the European Society of Clinical Microbiology and Infectious Diseases (EMESG)

## Abstract

**Background:**

Bacterial meningitis in children causes high rates of mortality and morbidity. In a recent clinical trial, oral glycerol significantly reduced severe neurological sequelae in paediatric meningitis caused by *Haemophilus influenzae *type b, and a tendency towards a benefit of adjunctive glycerol was seen in pneumococcal meningitis.

**Methods:**

Here we examined the effects of glycerol in pneumococcal meningitis of infant rats and adult mice. All animals received ceftriaxone, and glycerol or placebo. Brain damage, hearing loss, and inflammatory parameters were assessed.

**Results:**

Clinically and by histopathology, animals treated with glycerol or placebo did not differ. While both groups showed equally high levels of matrix metalloproteinase-9 at 24 h after infection, a significant difference in favour of glycerol was observed at 40 h after infection. However, this difference in matrix metalloproteinase-9 in late disease did not result in an improvement of histopathologic parameters.

**Conclusion:**

No benefit of adjunctive glycerol was found in these models of pneumococcal meningitis.

## Background

The outcome of pneumococcal meningitis (PM) in children has essentially not improved since the introduction of antibiotics [1]. A major cause of neurological complications is the excessive inflammatory reaction of the host [2,3]. To diminish this reaction, dexamethasone (DXM) is advocated as adjuvant therapy to antibiotics.

Secondary to the inflammatory reaction, brain oedema and increased intracranial pressure are life-threatening events in PM. Hyperosmolar agents, such as glycerol (GLY), have been suggested to antagonize these processes [4]. Important new information arrived from a large (n = 654) randomized, prospective and double-blind trial in which intravenous DXM was put vis-à-vis oral GLY and placebo [2]; GLY reduced severe neurological sequelae significantly in *Haemophilus influenzae *type b (Hib) meningitis, whereas DXM did not. In PM, a positive trend for GLY was observed on death or severe neurological sequelae. Data on GLY in adults with PM do not exist. Evidently, additional studies are warranted to further elucidate the pros and cons of GLY in PM [2,3]. Here we examined the effects of adjuvant GLY in PM of infant rats and adult mice.

## Methods

### Infant rat model of PM

An established model of infant rat PM was used [5,6]. Eleven days old Wistar rats were infected by intracisternal injection of 10 μl saline containing 1.4 × 10^6 ^± 5.3 × 10^5 ^colony forming units (cfu)/ml *Streptococcus pneumoniae *serotype 3 (n = 34), which has been isolated from a patient with invasive disease [7]. Control animals were injected with the same volume of sterile saline (n = 8). Animals were weighed and their clinical status was assessed at 18 and 40 h after infection [5,6]. Cerebrospinal fluid (CSF) was obtained at 18 h after infection by intracisternal puncture and 5 μl were cultured quantitatively [5]. Additional CSF was obtained, when possible, 24 h and 40 h after infection to assess the levels of matrix metalloproteinase (MMP)-9.

When starting the treatment at 18 h after infection, the animals were randomized to receive either 50 μl GLY 60% per os (n = 17 for infected and n = 4 for control animals) or 50 μl placebo (carboxymethylcellulose 2%) per os (n = 17 for infected and n = 4 for control animals) at 18 h, 24 h, 30 h and 38 h after infection. This corresponds to the dosing used in the clinical study [2]. Antibiotic therapy was started at 18 h after infection with 100 mg/kg body weight ceftriaxone subcutaneously administered to all animals twice daily. At 40 h after infection, the animals were sacrificed with an overdose of pentobarbital and brains were dissected.

All animal studies were approved by the Animal Care and Experimentation Committee of the Canton of Bern, Switzerland.

#### Assessment of MMP-9 levels in CSF

Levels of MMP-9 were assessed by gel zymography as previously described in detail [5,7]. The levels of the inducible MMP-9 were expressed as the percentage of the constitutively expressed MMP-2 [7].

#### Histopathology

For the assessment of an effect on acute brain damage a higher inoculum (4.0 × 10^6 ^± 0.0 cfu/ml, n = 56) was used in separate experiments. The histological assessment of brain damage was performed as previously described [5].

### Adult mouse model of PM

The mouse model used is well established [8]. Meningitis was introduced in C57BL/6 mice by injection of 15 μl of a bacterial suspension containing 10^7 ^cfu/ml of *Streptococcus pneumoniae *D39 into the cisterna magna under anaesthesia. At 18 and 24 h after infection, animals were examined clinically, randomized for 250 μl GLY 85% per os (n = 8) or placebo (carboxymethylcellulose 2%) (n = 8) and treated with ceftriaxone (100 mg/kg intraperitoneally). Effective administration of GLY was documented by an increase in serum osmolality in comparison to placebo treated animals (mean increase 6%). CSF was sampled by puncture of the cisterna magna. All mouse experiments were approved by the government of Upper Bavaria, Germany.

#### Determination of hearing thresholds

Hearing thresholds were determined by measuring auditory brainstem responses in mice as described previously [9]. The lowest stimulus intensity that elicited auditory brainstem responses was considered the hearing threshold.

#### Histology of the inner ear

The histological assessment of inner ear damage was performed as described previously [9]. Seven μm mid-modiolar sections of mice temporal bones were deparaffinized, re-hydrated, and stained with Mayer's haematoxylin and eosin.

### Statistical analyses

Statistical analyses were performed using Prism (GraphPad Software, San Diego, CA USA) or SYSTAT. Median [range] is presented for not normally distributed data while mean ± SD is given for normally distributed data. Kruskal-Wallis test was used to compare three or more groups with not normally distributed data. Mann-Whitney and unpaired t test was used for not normally and normally distributed data, respectively. Survival curves were analyzed with the Log-rank (Mantel-Cox) Test. *P *< 0.05 was considered statistically significant. Animals that died unobserved or were euthanized for ethical reasons were excluded from all analyses.

## Results

### Infant rat model

#### Clinical parameters of the disease

18 h after infection, all infected animals showed high bacterial load in the CSF. The bacterial load of animals treated with placebo vs animals treated with GLY did not differ (2.9 × 10^7 ^[4.0 × 10^6 ^- 7.0 × 10^7^] cfu/ml for placebo vs 1.2 × 10^7 ^[6.0 × 10^6 ^- 1.1 × 10^8^] cfu/ml for GLY; *P *= ns). The activity score (4.0 [4.0-4.0] for placebo vs 4.0 [4.0-4.0] for GLY at both time points) and weight loss (18 h after infection: 0.1 [-1.0-1.5] g for placebo vs 0.1 [-0.8-1.9] g for GLY; *P *= ns and 40 h after infection: -1.5 [-3.3-1.1] g for placebo vs -1.1 [-2.7-3.6] g for GLY; *P *= ns) did not differ between the treatment groups. Weight of control vs infected animals was significantly different (18 h after infection: 1.8 [0.7-2.7] g for controls vs 0.1 [-1.0-1.9] g for infected animals, *P *< 0.001 and 40 h after infection: 4.9 [1.2-6.4] for controls vs -1.2 [-3.3-3.6] g for infected animals, *P *< 0.001).

#### Assessment of MMP-9 levels in CSF

**Figure 1:**
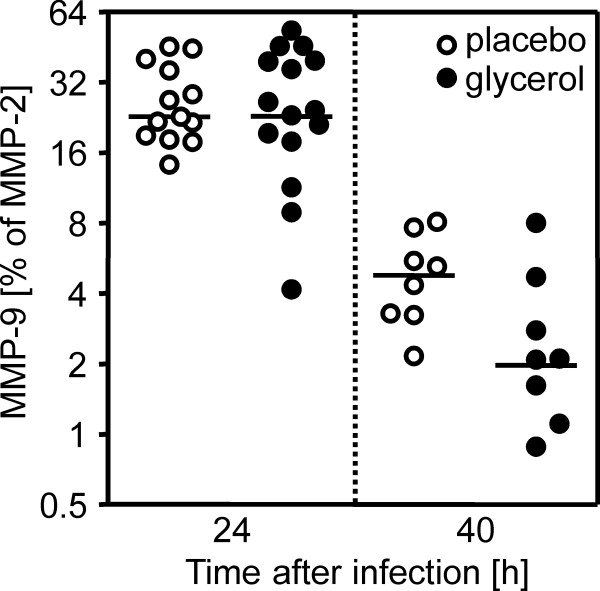
**MMP-9 levels in cerebrospinal fluid of rats with meningitis treated with placebo or glycerol**. The amounts of MMP-9 did not differ in the acute phase of the disease at 24 h after infection. Subsequently, the levels decreased approximately 10-fold in both treatment groups. At 40 h after infection glycerol-treated animals showed significantly (P < 0.05) lower levels of MMP-9 than placebo-treated animals (Log_2 _scale).

At 24 h after infection the levels of MMP-9 did not differ between the placebo- and the GLY- treated animals (Figure 1). After that time-point, MMP-9 levels decreased significantly from 23% to 5% and from 23% to 2% for the placebo and GLY group, respectively (*P *< 0.001 for placebo and GLY animals). The MMP-9 levels of the GLY-treated rats were significantly lower than in the placebo group 40 h after infection (*P *< 0.05; Figure 1).

#### Histopathology

Among the animals infected with the low inoculum, 27% showed apoptosis in the hippocampus and 42% showed cortical damage. No difference was found between the control and infected animals (Kruskal-Wallis test: *P *= ns for apoptosis and cortical damage). When the higher inoculum was used, 91% of the infected animals showed apoptosis and 62% cortical damage (Kruskal-Wallis test: *P *< 0.01 for apoptosis, *P *< 0.05 for cortical damage). Experiments with the high inoculum showed high mortality (60% for placebo vs 65% for GLY; Log-rank test: *P *= ns) which reflects a more severe disease. Adjuvant GLY (n = 11) vs placebo (n = 10) did not have an effect on apoptosis (0.5 [0.1-1.1 for placebo vs 0.6 [0.0-1.8] for GLY, *P *= ns) or cortical damage (1.0 [0.0-8.1] for placebo vs 0.3 [0.0-4.2] for GLY,*P *= ns).

### Adult mouse model of PM

At 18 h after infection, all animals showed clinical signs of infection by assessment of clinical score, weight loss, and a reduction of explorative activity. No difference in mortality was observed as the survival rate was 5/8 animals in the placebo and 4/8 animals in the GLY group (*P *= ns). Nor were differences observed in the clinical score (7.0 ± 2.6 for placebo vs 9.0 ± 2.9 for GLY, *P *= ns), explorative activity (16.6 ± 11.6 fields/2 minutes for placebo vs 10.0 ± 0.8 fields/2 minutes for GLY, *P *= ns), or weight loss (-17.5 ± 3.3% for placebo vs -18.7 ± 5.0% for GLY, *P *= ns). Measurements of brain albumin concentration reflect the blood brain barrier (BBB) breakdown. The concentration in brain lysates of uninfected animals was 44.0 ± 4.0 ng/ml and was increased in infected mice without differences between GLY-treated and placebo-treated mice (433.8 ± 262.0 vs 338.5 ± 187.7 ng/ml brain lysate). Moreover, CSF cell counts were similar between both groups (5700 ± 3381 cells/μl for placebo vs 5850 ± 714 cells/μl for GLY, *P *= ns).

The hearing thresholds were elevated in all infected animals and did not differ between the treatment groups. Similarly, histological differences in the inner ear were not observed. Granulocytic infiltration occurred equally in the perilymphatic space of the treated and non-treated animals.

## Discussion

The potential effects of adjuvant GLY in PM were evaluated in infant and adult rodent models of PM by assessing clinical, histopathological and functional outcome. Since GLY did not show an advantage over placebo in these parameters, this observation may partially explain why GLY did not significantly improve the outcomes of PM in children. In contrast, in Hib meningitis there is good evidence for using GLY, because it reduces severe neurological sequelae [1,2].

The aim of this study was to evaluate the effect of adjunctive therapy with oral glycerol in experimental pneumococcal meningitis in children and adults, using an infant rat and an adult mouse model, respectively. A direct comparison between glycerol effects on rats and mice was not the goal of the study and, thus, variations between the models should not affect the key issues of the study. Both models that were used are well-defined and have been optimized to develop the typical clinical, functional and histopathological features of pneumococcal meningitis in the respective age group. In the infant rat model, we regularly found clinical signs of meningitis, CSF pleocytosis, and histopathologic alterations such as apoptotic hippocampal damage. While in the adult model, we found clinical signs of meningitis, CSF pleocytosis, blood brain barrier damage, and hearing loss with cochlear damage. In the adult mouse model, we investigated hearing loss, cochlear damage, and the clinical condition (clinical score and motor activity) with a main focus on hearing loss and cochlear damage. Earlier studies showed that brain damage including hippocampal apoptosis is not a consistent finding in the adult mouse model but is regularly seen in the infant rat model [8]. Therefore, brain damage was not assessed histologically in the adult mouse model in this study. With respect to the drug dosage, we performed studies using the same glycerol dosage (10 mg/kg body weight per os, bid) in both models. However this led to excessive and early mortality in infant rats which precluded the assessment of apoptosis. These observations show exemplarily that models of infectious diseases need to be adapted for the assessment of clinically relevant outcome measurements.

Three clinical trials evaluated the effect of oral GLY on hearing impairment in bacterial meningitis [2,4,10]. A statistical difference for severe hearing impairment (> 80 dB) in GLY vs no GLY recipients was shown in one study [4] but has not later been confirmed [2,10], which fits the negative results of our adult mouse model examining hearing loss 40 h after infection. Since cochlear inflammation, a key factor for subsequent cochlear damage, appeared similarly in both treatment groups, it seems unlikely that a long-term benefit of GLY on hearing loss was missed in our study.

Previously, damage in the hippocampal dentate gyrus after GLY administration was found in a rabbit model of PM; GLY increased apoptosis, while placebo did not [11]. The present study does not support that finding, but did not show a protective effect either. No protective effect of GLY against meningitis-induced cortical damage or BBB breaching was observed either. Combined, our study does not provide evidence for a neuroprotective action of adjuvant GLY therapy in PM.

MMPs degrade the extracellular matrix including the subendothelial basement membrane which forms the BBB and are considered as effector molecules of BBB breakdown and leukocyte extravasation in bacterial meningitis [7]. Regarding bacterial meningitis, MMP-9 seems especially interesting, because its concentration increases in CSF and the brain tissue of patients and animals in an experimental setting [6,12-14]. MMP antagonism in experimental models has reduced brain damage and BBB disruption [6,12-14].

In this study, the placebo and the GLY groups showed equally high levels of MMP-9 24 h after infection. Subsequently, the levels decreased approximately 10-fold in both treatment groups until 40 h after infection when the GLY group showed significantly lower concentrations as compared with the non-GLY group of animals. Despite this divergence in MMP-9 levels in late disease, no difference was observed in the extent of neuronal damage or BBB disruption. It is conceivable that neuronal and vascular damage develop in the early phase of disease, when the MMP-9 levels are equally high. A reduction of MMP-9 at a later stage is probably too late to influence the already launched detrimental process.

## Conclusion

We detected no benefit of adjunctive GLY in an infant and adult animal model of PM. Thus, due to the limited number of tools besides antimicrobials to improve the prognosis of PM, the search for new approaches needs to be continued.

## Competing interests

The authors declare that they have no competing interests.

## Authors' contributions

CB and MK carried out the experiments, analysed the data and drafted the manuscript on the infant rat and adult mouse model, respectively. MK, DG, UK and MWi participated in the design of the study and the data analysis. MWe carried out the osmolality measurements. HP and UK participated in writing of the manuscript. SLL conceived and coordinated the study, participated in study design, data analysis and in writing of the manuscript. All authors read and approved the final manuscript.

## Pre-publication history

The pre-publication history for this paper can be accessed here:

http://www.biomedcentral.com/1471-2334/10/84/prepub
